# Adolescent's Willingness to Adopt a More Plant-Based Diet: A Theory-Based Interview Study

**DOI:** 10.3389/fnut.2021.688131

**Published:** 2021-08-30

**Authors:** Remco C. Havermans, Geert Rutten, Dimona Bartelet

**Affiliations:** ^1^Laboratory of Behavioural Gastronomy, Center for Healthy Eating and Food Innovation, Maastricht University Campus Venlo, Venlo, Netherlands; ^2^Chair Group Youth Food and Health, Maastricht University Campus Venlo, Venlo, Netherlands

**Keywords:** adolescents, food choice, attitude, plant-based diet, taste

## Abstract

A plant-based diet is more sustainable, and research suggests that adolescents obtain health benefits from adopting a more plant-based diet (e.g., improved weight control, increased cardiovascular health). However, it is still unclear what factors promote/hinder their intention to attain such a dietary habit. The aim of the present study was to examine factors affecting adolescents' willingness to consume more plant-based foods. In a theory-based interview study, using the Reasoned Action Approach as framework, adolescents were interviewed concerning their reasons for their (un)willingness to adopt a more plant-based diet. A total of 11 adolescents (15/16 years old; 7 girls, 4 boys) were recruited and interviewed at two secondary schools in the Dutch Province of Limburg, the Netherlands. None of the interviewees reported being vegan/vegetarian/flexitarian. The interviewed adolescents overall expressed little interest in adopting a more plant-based diet. They appeared to have little knowledge of what would comprise a more plant-based diet and showed a lack of awareness of the benefits for personal and planet health. Further, most participants indicated lacking skills to prepare plant-based meals. Despite that, they felt confident they would be able to consume a much more plant-based diet for a definite period. Most importantly, the low intention to consume more plant-based food options was explained by the perceived (or expected) poor taste of these foods. We conclude that education on the plant-based diet (i.e., increasing awareness of the benefits, and skills to procure or prepare a plant-based meal) might increase both knowledge and plant-based food familiarity. The latter being important as food familiarity is key in promoting its acceptance.

## Introduction

Research suggests that as adolescents gain independence, they make fewer healthy food choices (1, 2). Indeed, few adolescents adhere to a healthy diet (3). This is problematic as dietary habits established during adolescence are likely to influence long-term eating behavior (4, 5). The adolescent diet typically comprises foods high in fat, sugar, and salt (3), and the intake of plant-based foods such as fruits, vegetables, nuts and seeds generally does not meet dietary recommendations with a large proportion of adolescents not even consuming fruits and vegetables on a daily basis (6). Adolescents in Europe also tend to consume much more meat than is recommended (3), which is problematic as popular processed meat products like hamburgers and sausages contain high amounts of saturated fat and salt. Therefore, various scholars have argued that healthier eating might best be achieved with the adoption of a more plant-based diet; that is, meals mainly centered on nutrient-dense plant-based foods (vegetables, fruits, whole grains, legumes, seeds, and nuts), with reduced amounts of animal source foods (especially red and processed meats) (7–9).

A diet that is mainly plant-based, as opposed to a more meat-based diet (7), is relatively low in saturated fat, nutritionally adequate, and conveys various health benefits (e.g., lowered risk of developing obesity, protection from cardiovascular disease, type 2 diabetes, and various forms of cancer) (10, 11). Moreover, as livestock significantly contributes to greenhouse gas emissions, reduced consumption of animal products in favor of a more plant-based diet holds the promise of being more environment-friendly. It should be noted that not all plant-based food options are necessarily healthy. For example, plant-based meat replacements are highly processed products that are energy dense and often contain too much salt. Even so, a recent study demonstrated that the consumption of these more highly processed plant-based meat replacers was still significantly associated with a lowered risk of coronary heart disease (9). The question then is how to stimulate the adoption of a more plant-based diet, particularly in adolescents.

Understanding and effecting behavior change requires theory. To date, the most popular approach to behavior change is the Reasoned Action Approach, an integrative conceptual framework that helps effectively predict and explain a wide variety of behaviors with only a limited set of constructs. Briefly, any behavior is thought to be predicted chiefly by intentions which in turn are informed by the attitude toward the behavior (positive/negative), the perceived social norm to engage in the behavior, and perceived behavioral control regarding the behavior (12). The aim of the present study was to examine adolescents' intention to (not) adopt a more plant-based diet, using the Reasoned Action Approach (henceforth RAA) as theoretical framework (described in more detail below in the Methods section) in trying to identify the relevant determinants of said intention (13).

## Methods

The present study was preregistered on 24 February 2019, at the Open Science Framework (OSF; https://osf.io/c5pws/).

### Sampling and Ethical Considerations

Adolescents were approached by their teachers at two secondary schools in the Province of Limburg (one in Nederweert – a town adjacent to the city Weert – and the other in Reuver – a village close to the larger city Venlo), in the Netherlands. Limburg borders Germany and Belgium, covering an area of over 2000 km^2^ with a population size of ~1.2 million citizens. In the Netherlands, children attend secondary school at age 12. Dutch secondary education distinguishes three main streams: VMBO (Voorbereidend Middelbaar Beroeps-Onderwijs – *preparatory mid-level vocational education*), HAVO (Hoger Algemeen Voortgezet Onderwijs – *higher general continued education*), and VWO (Voorbereidend Wetenschappelijk Onderwijs – *preparatory science education*). VMBO (4 years of secondary education) prepares for vocational training and corresponds to the average educational level in the Netherlands. We purposively sampled 15/16-year-old VMBO pupils as most Dutch adolescents attend this educational stream and thus represent a wide range in cultural and socio-economic background. Our focus on this narrow age range in the present study was also informed by the fact that at this age most adolescents have a job (outside school) which allows them to increasingly start making their own food choices. Note however, that we did not consider employment status as an inclusion criterium. The inclusion of 15/16-year-olds also allowed us to recruit among the students in the final year of their secondary education. These students have a more flexible teaching schedule, which allowed us more flexibility in planning interviews. Apart from selecting participants from a definite age range, we aimed for diversity of sex. Participants were not selected on the basis of their educational achievements. Note that we did not exclude or include participants based on their current eating pattern, allowing for the possibility that some participants might already have switched to a complete/largely plant-based diet.

The study protocol was reviewed and approved by a local review board (#ERCIC_117_23_01_2019). All interviewees were informed beforehand on the purpose of the study and provided written consent. The participants were informed that we aim to learn more about adolescents' food preferences, especially considering plant-based food choice, and their reasons for eating certain foods but not other foods. The parents of the participants below 16 years also had to provide written consent. All participants (and parents) were made aware that interviews would be recorded and transcribed, and that data would be handled confidentially by storing recordings on a password protected data server accessible only by the involved researchers. Participation was purely voluntary; no incentives were provided.

We determined a minimally sufficient sample size for achieving data saturation based on prior experience and recommendations from literature (14). The aim was to interview a total sample size of 10–20 adolescents. We thus initially interviewed 7 participants and then used a stopping criterion of no new information acquired for at least three subsequent interviews. In total, we interviewed 11 15/16-year-olds (7 girls; 4 boys). The anonymized transcribed interviews are shared on the OSF (https://osf.io/c5pws/).

### Data Collection and Reasoned Action Approach

Individual, in-person semi-structured interviews (18–30 minutes long) were conducted in between regular classes in a separate, quiet room at the attended school. Interviews were recorded with a digital voice recorder (Philips DVT1110) in March and April 2019. We created an interview guide using RAA as theoretical framework. On the basis of the literature (2, 13, 15) and given the target behavior and sample we selected background, proximal, and moderating factors with regard to adopting a more plant-based diet. The full interview guide (translated from Dutch) is shared on the OSF (https://osf.io/c5pws/).

According to the RAA (12), the immediate antecedent of a behavior is intention (i.e., the readiness to perform a certain act) which is informed by behavioral beliefs, normative beliefs, and control beliefs such as self-efficacy. RAA has proven to be a very useful framework for qualifying the intention toward specific health behaviors, thus providing relevant information on how to effectively promote healthy choices – including healthier food choice in adolescents and young adults (13, 15–17). With regard to food choice in young adults, Wyker and Davison (13) found that college students' behavioral beliefs (e.g., how good, pleasant, beneficial, and enjoyable a plant-based diet would be) were the strongest predictor for their willingness to adopt a plant-based diet. Stevenson and colleagues (15) noted that adolescents generally report a strong preference for high energy dense foods. In their study, the adolescents also reported being very little involved in food purchase and preparation. Furthermore, healthy eating was considered an unpleasant activity. Ensaff and colleagues (2) recently also found that adolescents consider healthy plant-based foods as being less tasty than animal-based products, lack knowledge of plant-based diets, and are largely unaware of the potential benefits of consuming more plant-based foods. How these various beliefs however may inform the intention to adopt a more plant-based diets in adolescents is still unclear.

**Figure 1:**
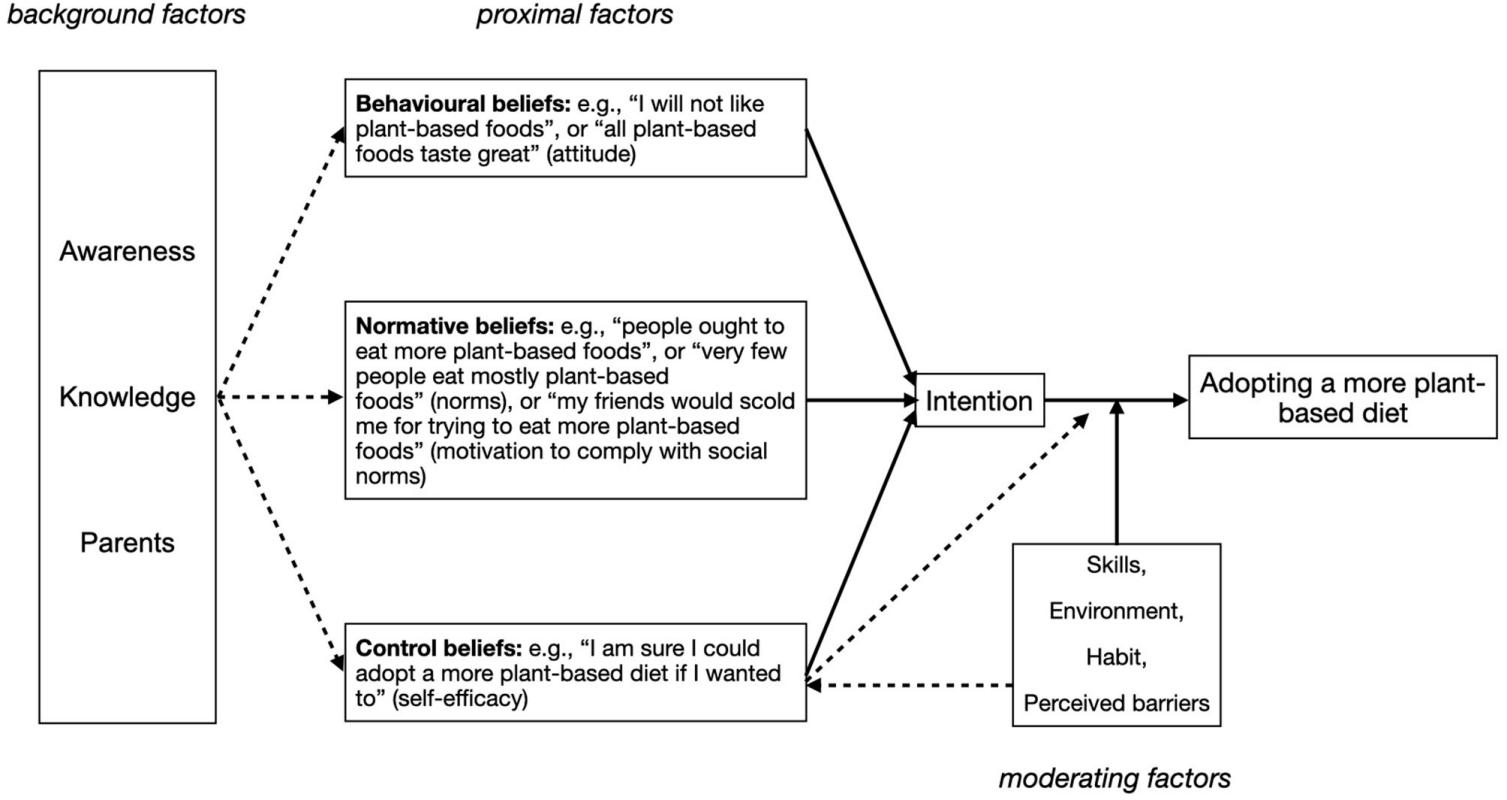
The reasoned action approach (RAA) as applied to examining adolescents' willingness to adopt a more plant-based diet. Choosing more plant-based food options is defined as the target behavior. Behavioral, normative (including the motivation to comply with norms), and control beliefs depicted as proximal factors relate to the intention to choose more plant-based food options. Parents, as well as adolescents' degree of awareness of the benefits of eating more plant-based foods and their knowledge concerning a plant-based diet were identified as (potentially) informing these beliefs. Skill in preparing/procuring a plant-based meal, environmental availability of plant-based food options, habits impacting the adoption of a more plant-based diet, and perceived barriers toward adopting a more plant-based diet were considered potential moderators.

As shown in Figure 1, in applying RAA for the purpose of this study we narrowed down behavior to “adopting a more plant-based diet”. The willingness to do so (intention) is informed by proximal factors. These are typically beliefs that refer to: (1) the adolescents' attitude toward adopting a more plant-based diet (behavioral beliefs; e.g., asking “Do you think you might enjoy the taste of certain plant-based foods, of meat replacers for example?”); (2) motivation to comply with perceived social norms relating to the adoption of a more plant-based diet (normative beliefs; e.g., asking “Do you find it important what your friends think about what you choose to eat?”); (3) perceived behavioral control (or self-efficacy) with regard to adopting a more plant-based diet (control beliefs; e.g., asking “Do you believe you would be able to recognize plant-based foods in the supermarket to compose a lunch with?”). These beliefs in turn are potentially informed by background factors; that is, individual factors (e.g., personality), or social factors (e.g., education, culture), or information factors (e.g., knowledge and media). Fishbein and Ajzen (12) state that there can be a large number of factors that might affect different beliefs. For the application of the framework, it is important to identify those factors that likely play such a role. We identified parents (as models for dietary behaviors, as food providers, as caregivers, determiners of socio-economic status, and influencing values and general attitudes), awareness of the benefits of adopting a more plant-based diet (e.g., for personal health, animal welfare, and environmental burden), and knowledge on plant-based foods (what is a more plant-based diet and where can one purchase plant-based foods or meals, and how can one prepare a more plant-based meal) as background factors of interest.

The intention to eat more plant-based foods is not synonymous to the actual behavior. The link between intention and actually adopting a more plant-based diet can be moderated by various factors, such as the degree in skill in preparing a healthy plant-based meal, or the availability of plant-based food options in the environmental context of the adolescent.

### Photo Elicitation

We employed a photo assignment to facilitate conversation, to create a rapport with the participant, and to better contextualize participants' knowledge regarding a plant-based diet in the later interviews. The main purpose of the assignment was to elicit information from the participants by involving participant-generated photos (18).

Before the interviews we gave the participants the assignment to take 2–6 pictures of their lunch, of healthy foods, of plant-based foods/meals, and of sustainable foods by using their smartphone. Participants were also allowed to search and download relevant pictures from the internet. They were asked to email their photos within 2 weeks after the assignment was provided. A printed card with a summary of the assignment was given to each participant as a reminder. The photos were used to support starting a conversation and discussion with the participants asking them for instance to point out which elements of their lunch they believe are plant-based.

### Coding and Analysis

All digitally recorded interviews were transcribed verbatim and imported as text documents in ATLAS.ti (Scientific Software Development GmbH, Berlin, Germany), a qualitative analysis program. We used a content analysis with a directed approach. This approach is appropriate when theory or prior research exists about a phenomenon that is incomplete or would benefit from further description (19). The RAA informed the development of a pre-structured coding scheme. Additional flexible codes were used for other prominent topics that were derived from the data and did not match with existing codes.

Two research assistants first coded all transcripts independently, then compared coding and resolved discrepancies. One researcher (RCH) then reread all coded transcripts and supplemented the pre-structured coding scheme to create a coding manual that was finalized after feedback from a second researcher (GR). In the draft coding manual for example, the background factors were referred to as distal factors and intention was defined as a motivation rather than a readiness or willingness to adopt a more plant-based diet. This was corrected when finalizing the coding manual, which was then used to recode all transcripts. The final coding manual – including the interview guide, the initially generated codes and written feedback on the draft coding manual – is publicly shared on the OSF (https://osf.io/c5pws/).

In order to maintain reflexivity, the team engaged in bi-weekly discussions throughout the duration of the project, discussing methods, analysis and interpretation of results. These discussions were informed by our individual differences in scholarly background in developmental psychology (DB), public health (GR), and behavioral nutrition (RCH). Further, the first author presented findings and tentative conclusions for further discussion with peers at a wider department research meeting. We conducted these discussions with the intent to limit the threat of our conclusions being particular to the perspective of just one researcher (20).

## Results

Results were organized in line with the theoretical model we used to qualify adolescents' willingness to adopt a more plant-based diet, distinguishing background factors (i.e., awareness of benefits related to plant-based diet, knowledge of what comprises a plant-based diet, and parental influence), proximal factors (i.e., behavioral beliefs, normative beliefs, and control beliefs), and moderating factors (i.e., environment, eating habits, perceived barriers, and skills pertaining to the preparation of a plant-based meal) affecting the adoption of (or intention to adopt) a more plant-based diet (see Figure 1 above). Note that participants' quotes below illustrating these factors were translated from Dutch by one of the authors (RCH).

### Background Factors

Ten participants articulated no or only low awareness of the benefits of adopting a more plant-based diet, occasionally noting the potential effect on the environment or the potential benefit for one's health.

*I've heard that if people would eat a bit less meat, it would benefit the environment or something* (female, 16).

Most participants (*n* = 10) expressed little certainty regarding their knowledge on what food products are plant-based or where to procure these.

*I think cucumbers are plant-based because they're from plants* (female, 16),

*Yeah, I wouldn't know if all supermarkets have separate shelves for that* [plant-based products] (female, 16).

Also, two participants expressed interest in learning more about adopting a more plant-based diet.

*Yeah, I think that's interesting…biology and stuff…I think it's an interesting topic* (male, 15).

Most participants were reluctant to explicitly acknowledge the influence of their parents on their eating behavior. Nonetheless, all participants clearly expressed varying ways in which their parents affect their food choice and food intake. Parents often had a strong opinion on plant-based dietary options, conveyed a certain norm of what comprises a healthy, nourishing meal, and varied in their competence to prepare such meals. Furthermore, at home, parents largely controlled what foods were available. As many interviewed participants noted, parents often actively stimulated the consumption of certain plant-based foods, fruits mainly. Some of the interviewed adolescents indicated that their parents were consciously trying to eat meat less frequently and adopt a more plant-based diet, whereas other participants strongly believed their parents would condone but not support them if they would want to adopt a more plant-based diet.

*My dad always says, “I'm going to take life more seriously”, and then he still visits the pub every day. My mum and her boyfriend are always super healthy. So, there is lots of healthy food there. Much less so at my dad's* (male, 16).

*With every family supper we have vegetables, meat, and potatoes or rice* (male, 15).

### Proximal Factors

As regards their *attitude*, nine participants expressed certain behavioral beliefs concerning expected outcomes of eating more plant-based foods and fewer animal products. The (expected) hedonic taste and the appearance of food (e.g., color, presentation, packaging) appeared to play a very important role in their attitude toward plant-based food products. This concerned not necessarily plant-based food options specifically, but any food in general.

*Actually, it's just the taste* [of any food]. *If I don't like it, I don't want it* (female, 16),

*As long as it* [i.e., any food] *looks palatable. I don't buy food because it's sustainable. I buy it because it looks good* (female, 15).

Related to *social norm*, only three participants indicated that they knew someone who consciously eats more plant-based foods (e.g., a vegan or a vegetarian). Most participants stated that they did not know anyone who does not eat animal products (meat particularly) every day. In their opinion eating meat was the norm.

*I don't think so* [regarding the question if she knew many people who are vegetarian or try to consume only a limited number of animal-based products] *because everyone eats that, nearly everyone eats meat* (female, 16).

Further, most participants (*n* = 10) expressed little motivation to comply with important others (notably peers, siblings, parents) as regards their food consumption.

*I don't care that much about what other people eat* (male, 16).

A large majority of nine participants expressed high *self-efficacy* toward eating fewer animal products and more plant-based foods if deemed necessary. However, they generally failed to indicate how they might accomplish that transition. Self-efficacy of the remaining two participants toward achieving the transition to a more plant-based diet was low.

*I could try* [eating more plant-based meals] *but I would fail. I know that already. After a week or two, I'd no longer want any more lettuce* (male, 15).

### Intention to Adopt a More Plant-Based Diet

Only two participants expressed a clear intention to consume fewer animal products, whereas two other participants expressed some willingness to try reducing their meat or dairy consumption. The majority of participants (*n* = 7), though, expressed no or little intention to (try) adopt a more plant-based diet.

*Yeah, planning to, I don't know. I could try but yeah, usually it won't happen because meat is still very nice* (female, 15).

### Moderating Factors

Participants were asked about the influence of the *environment*, about their *nutritional habits* and about their *perceived barriers* and *skills* toward plant-based food consumption. *Six* participants noted that many animal food products, high energy dense snack foods mainly, are very cheap and readily available in nearby stores or even at school.

*Usually, during school breaks, I go to the store to buy a* […] *Tok Tok* [chicken nugget] *sandwich* (male, 16).

Six participants remarked that they do not give their eating behavior much thought. Food choice seemed largely habitual and based on daily routines. Eating unhealthy foods is often prompted by salient environmental cues, which was recognized by one participant as a likely barrier for transitioning to a more plant-based dietary pattern.

*When I would see someone else eating a hamburger, I would think “Oh, I want that too”. That makes it hard because I can't tell everyone: “I don't want you to eat meat when you're around me”* (female, 16).

Only one participant indicated to possess the skills to prepare a full plant-based supper because he used to work at a wok restaurant, whereas none of the other participants had the skill to cook a meal at all. One participant adamantly noted having neither the skill nor the desire to cook.

*Cooking is really super tedious. You have to wait until it is done and then you need to flip it over and it collapses. And it always ends up burnt and sticky even when I stir. So yeah. No*. (female, 16).

### Current Behavior

Considering the participants' current behavior in relation to adopting a more plant-based diet, all participants claimed that they regularly eat fruits and vegetables, though six participants admitted that they do not eat fruits and/or vegetables every day. Six participants noted that they regularly (nearly every day) ate crackers, whole wheat cookies, or rice cakes with their lunch or as a snack, but no participants mentioned nuts/seeds as a regularly consumed plant-based food with one participant explaining she has a nut allergy. Notably, three participants considered nuts (including peanuts) only as a meat replacer. All participants indicated eating meat (nearly) every day. Six participants even noted that they eat a lot of meat every day, but two participants stated that they consciously attempt to limit the amount of meat they eat with their meals (typically supper). Only one participant indicated regularly consuming a dairy replacer (i.e., chocolate flavored soymilk).

## Discussion

In the present study, we examined 15–16-year-old adolescents' intention to adopt a more plant-based diet, applying the RAA as framework. According to this adapted framework, there are distal background factors and more direct proximal factors informing the intention to adopt a more plant-based diet. In terms of background factors, we found that the interviewed adolescents expressed little knowledge of what products typically comprise a more plant-based diet, or where to procure these foods. They also stated little awareness on why adopting a more plant-based diet might be beneficial to their health or better for the environment. Parents also seemed to still play an important role in directing and facilitating food choice in these adolescents. Apart from background factors, the relation of proximal factors (i.e., behavioral, normative, and control beliefs) with the intention to adopt a more plant-based diet were assessed. Importantly, most of the interviewed adolescents stressed the importance of taste. If plant-based foods were tasty and would look palatable they would be inclined to try it (a behavioral belief). The consumption of animal-based products, notably meat products, was considered the norm by most participants (normative belief), but the participants believed they are not influenced by such norms or (more directly) by peer behavior when it comes to making food choices. Most participants expressed the belief that they could transition to a more plant-based diet (control belief), but most participants also stated they do not wish to do so (intention). As stated above, the lacking intention to adopt a more plant-based diet was often explained as an unwillingness to give up pleasure derived from consuming animal-based products (particularly meat). The adolescents' environment (home, stores, school) generally offered many opportunities for unhealthy food choices. Food cues from the environment would often evoke the urge to eat. The interviewed adolescents also professed little food preparation skills. These are all factors that may moderate and lower the chances of adopting a more plant-based diet (as defined in the current study) even when an adolescent is willing to do so. Apart from these findings, we noted that adopting a more plant-based diet was often interpreted by the adolescents as ‘eating less meat’ specifically or equated with becoming a vegetarian (or vegan even), which is in line with recent findings from a large survey study (21).

### Willingness to Adopt a More Plant-Based Diet

An important driver of the participants' intention to eat anything in general appeared to be taste. When participants believed that plant-based foods or meals do not taste as good as (or worse) than animal-based foods, they expressed a reluctance to try it. Plant-based foods need to taste good and look tasty for these adolescents to consider trying more plant-based foods. This too is in line with previous research. For example, it was found that adolescents' demand for fast food meals is determined to a large degree by how tasty they believe it to be (22). Stevenson and colleagues (15) also found that, overall, adolescents tend to prefer high energy dense foods over less energy dense healthy foods such as fruits, vegetables, nuts and seeds. And they resist challenges to their food (dis)likes. Interestingly, the few adolescents in their study who considered themselves healthy eaters also reported being more interested in cooking. In the current study, all the adolescents, except for one reported having no skills to prepare/cook a meal. It would be interesting to learn whether training adolescents' cooking skills helps them in developing healthier food preferences.

Surprisingly, when asked directly the interviewed adolescents expressed a low motivation to comply to either their peers or parents on their food choice and intake, herewith indicating an absence of social influence on their nutritional behavior. Of course, it is possible that peer pressure and conformity is more relevant in the context of risk-taking behaviors. But in more indirect observational studies, adolescents are certainly influenced by their peers when choosing food or are at least sensitive to descriptive norms (23, 24). Perhaps adolescents are not acutely aware of being influenced by their peers, or adolescents feel it is more important to express their autonomy and independence (25). As a consequence, overt questioning may not be the best way to identify social influence on adolescents' (nutritional) behavior. It would be useful to administer standard questionnaires measuring adolescents' conformity disposition in future research (26).

In contrast to the participants' general assertion that they rarely comply to the behavior of others, all interviewed participants were clearly influenced by their parents' eating behavior and food choice. This is not a paradox, however. It is conceivable that adolescents reflect on their own eating behavior and food choices autonomously, still taking the opinion and values of their parents into account when doing so (27, 28).

Factors qualifying adolescents' intention to adopt a more plant-based diet, as suggested by the current study, as well as in previous studies, show considerable overlap with the research literature on reasons to adopt a more plant-based diet in adults. For example, when validating their Meat Attachment Questionnaire, researchers identified positive attitudes toward eating meat (e.g., enjoying its taste) as an important factor informing the willingness to adopt a more plant-based diet in a sample of Portuguese consumers (29, 30). The enjoyment of the taste of meat was also identified in a survey study among adult Finnish consumers as an important barrier toward adopting a more plant-based diet. Furthermore, and in agreement with findings from the current study, it has been found that low familiarity with plant-based food options and perceived difficulty in preparing plant-based meals are important barriers for adopting a plant-based diet (31). More recently, research among Danish consumers demonstrated that taste experience with plant-based meals can facilitate its acceptance (32).

### Strengths and Limitations

In the present study we employed individual interviews with adolescents. The use of RAA allowed us to discuss a predefined range of topics with the adolescents providing focus to the interviews. Reflexivity was acknowledged among the team members and maintained through frequent discussions within the team, but also with peers outside the research team allowing discussions on contesting or competing conclusions. We deliberately used RAA as a frame of reference as it is a broad theory of behavior frequently applied in public health research including the design of health interventions for adolescents (17). Nonetheless, it is not the only behavior theory we could have selected as a frame of reference and we acknowledge that a different theory (or set of theories) might have guided us to slightly different findings.

Further, the use of a photo elicitation assignment was intended to provide a concrete means to initiate discussions during interviews. The aim was also to elicit information from the participants by involving participant-generated photos. However, few participants adhered to the assignment as described above. Two participants admitted they simply forgot to make any pictures. Of the participants who did take pictures, only one participant submitted all the pictures as originally requested. Although we deem photo elicitation to be a worthwhile tool for initiating discussions, it of course fails to be of much use if participants generally do not adhere to the assignment. Interviews were not re-scheduled when participants indicated not having taken the pictures as was originally requested. The photo-elicitation assignment was intended to support discussion on adopting a more plant-based diet but not as a prerequisite for having such a discussion. In the absence of any pictures to discuss, adolescents were asked to describe their typical lunch as a starting point for the discussion instead. A photo-elicitation assignment may however have value beyond supporting a discussion. Indeed, when used as an integral research tool, it may not only help support discussion and provide rapport but also help to generate information more deeply (18). In that latter sense, our failure in having participants adhere to the photo elicitation task is clearly a limitation.

We did not employ different methods of data collection for triangulation. Apart from interviewing the adolescents we could have surveyed parents and teachers or obtained observations of food choice and eating behavior within school canteens. These forms of data might have informed and refined our final conclusions. Nonetheless, our results and conclusions do corroborate prior research. For example, the overall importance of pleasant taste (and not so much health benefits) in adolescents' food choice has been found in various studies (as described above) (2, 13, 15, 22, 29–32).

We discussed and explained what we think a more plant-based diet is at the start of each interview. We emphasized that a plant-based diet is not a vegetarian/vegan diet. It is fine to consume animal-based products such as meats, milk, cheese, butter, eggs, and honey, if the overall diet mainly comprises plant-based foods such as fruits, vegetables, nuts, and seeds. We were careful though to emphasize that not all plant-based foods are necessarily healthy foods, giving potato chips as an example. We wanted to prevent the interview to center solely around healthy vs. unhealthy food choice. Nonetheless, we did ask participants their opinions on vegan meals, meat replacers, and vegetarians, possibly leading some participants to interpret a plant-based diet as being a vegetarian/vegan diet. This is a potential limitation underscoring the need for triangulation in future studies.

Further, we did ask for participants' current eating behavior (mainly habits and food choice), but we did not obtain relevant background information on the interviewed participants, such as socio-economic status or employment status of the adolescents. Furthermore, the small sample of interviewees cannot be considered representative of the Dutch adolescent population. These are limitations regarding the examined constructs that needs to be addressed in future research.

## Conclusions

In sum, the present study suggests that adolescents show little intention to adopt a more plant-based diet which may be associated with having limited knowledge, awareness, and skills regarding a plant-based diet. For the interviewed adolescents, it was important that plant-based foods look and taste as good (and hence be as enjoyable) as animal-based food products. They have little experience and are not all too familiar with meat and dairy replacers. Merely increasing knowledge will not contribute much to changing adolescents' intention to consume more plant-based foods (33).

As repeat food exposure typically promotes acceptance and better liking for the exposed food (34), increasing plant-based food familiarity seems key in promoting its acceptance, especially in adolescents who (relative to adults) might still be food neophobic to some degree (35). For children in middle adolescence (14–16 years old), the school and home environment are the more relevant contexts in which such plant-based food familiarity and taste experience might be trained. When such training is mandatory, consuming more plant-based foods might also become the norm. And as far as such training in the form of nutrition education also involves teaching how to prepare and procure tasty plant-based foods, such an intervention may contribute to perceived behavioral control. Apart from physical environments, a social media setting may also be a viable context for educating and familiarizing adolescents with more plant-based food options (36). Of course, such an intervention would not address important background factors as the awareness of benefits of adopting a more plant-based diet. It would not directly alter parents' perceptions of adopting a more plant-based diet and it would not change the obesogenic environment the adolescent needs to navigate. Nonetheless, whether a nutrition education intervention, in either schools or online, can still increase adolescents' intention to adopt a more plant-based diet warrants further research.

## Data Availability Statement

The datasets presented in this study can be found in online repositories. The names of the repository/repositories and accession number(s) can be found below: https://osf.io/c5pws.

## Ethics Statement

The study protocol was reviewed and approved by the Maastricht University Ethical Research Committee of the Inner City Faculties (#ERCIC_117_23_01_2019). Written informed consent to participate in this study was provided by the participants' legal guardian/next of kin.

## Author Contributions

RH, GR, and DB designed the study (conception of the study, developing the research plan, and study oversight). DB conducted interviews with help of undergraduate students supervised by GR. DB and RH transcribed the interviews. GR and RH developed a coding manual and RH finally coded all transcribed interviews. RH wrote the manuscript. GR and DB read and provided feedback on a first draft and approved the final version of the manuscript.

## Conflict of Interest

The authors declare that the research was conducted in the absence of any commercial or financial relationships that could be construed as a potential conflict of interest.

## Publisher's Note

All claims expressed in this article are solely those of the authors and do not necessarily represent those of their affiliated organizations, or those of the publisher, the editors and the reviewers. Any product that may be evaluated in this article, or claim that may be made by its manufacturer, is not guaranteed or endorsed by the publisher.
